# Exploring Genetic Diversity: Optimizing Simple Sequence Repeat (SSR) Markers in *Crotalaria* for Enhanced Precision in Biodiversity Research

**DOI:** 10.1155/sci5/2409286

**Published:** 2025-06-01

**Authors:** Phenny Sharon Odhoch, Nancy L. M. Budambula, Felix Kiprotich, Joshua Kiilu Muli

**Affiliations:** ^1^Department of Biological Sciences, University of Embu, Embu, Kenya; ^2^Department of Biological Sciences, University of Kabianga, Kericho, Kenya

**Keywords:** biodiversity, Crotalaria, genetic diversity, polymorphic information content, simple sequence repeats

## Abstract

*Crotalaria* is a plant genus with more than 700 species of shrubs and herbs. Despite its potential economic importance, *Crotalaria* has received limited research attention; hence, there is limited information on its genetic diversity. Hence, there is need to establish its genetic diversity as a foundation for its conservation and breeding. The current study aimed to optimize and validate simple sequence repeat (SSR) markers polymerase chain reaction—conditions for the assessment of genetic diversity in *Crotalaria.* The genomic DNA of 31 *Crotalaria* accessions was extracted from 2-week-old leaves using a modified CTAB protocol and Quick-DNA Plant/Seed Kits (Zymo Research Corp) were used for recalcitrant samples. The samples were then amplified using the 29 SSR markers under the optimized conditions. The polymorphism information content (PIC) of the polymorphic markers was calculated to determine their effectiveness. This study determined that the optimal concentrations of dNTPs, MgCl_2_, and primers as 2.5, 2, and 5 mM, respectively, and the quantity of the DNA template was 1 μL, and the quantity of *Taq* was 0.125 μL in a 25 μL reaction mixture. The mean PIC value was 0.233, which shows that the markers were slightly informative. The marker PC004 was the most informative marker with the highest PIC value (0.605) and it detected the largest number of alleles despite being a hexanucleotide motif repeat. Its uniqueness augments its potential use in the assessment of genetic diversity. This study implies that the SSR markers designed and optimized for the study are significant for genetic diversity and population structure analysis of *Crotalaria* species and molecular verification of *Crotalaria* genotypes as well as other related genera. Besides, the results of the study form a basis for genetic improvement of *Crotalaria.*

## 1. Introduction

The genus *Crotalaria* is a member of the Family Fabaceae and the Subfamily Aboideau [1]. The genus is large and comprises approximately 702 species. Members of this genus are extensively distributed in the Southern Hemisphere [2]. About 543 species in the genus are distributed in subtropical and tropical Africa and Madagascar. The Fabaceae family has the largest number of indigenous vascular plants in Kenya with 93 species in the genus *Crotalaria* [3]. Plants in the genus *Crotalaria* have several uses including green manure, ethnomedicinal uses, silage, nitrogen fixation, use in weed control as a cover crop, ornamental plants, nematode control, and prevention of soil erosion [4]. Several species of this genus such as *C. juncea* have exhibited antimicrobial properties, making them a promising collection of plant products. Research has found that extracts from different parts of *Crotalaria* species, such as leaves of *C. capensis* and *C. madurensis*, the flowers and seeds of *C. pallida* and *C. juncea*, and the root of *C. burhia*, show antimicrobial activity against various bacteria, such as *Enetroccocus faecalis*, *Staphylococcus aureus*, *Salmonella typhimurium*, *Pseudomonas aeruginosa*, *Bacillus subtilis*, and *Bacillus cereus* [5]. Despite their industrial potential and economic importance, these species have received little research attention, thus information on their genetic diversity remains scanty [6]. Consequently, few genetic markers exist to facilitate the study of the plant's diversity.

Genetic diversity of crops plays a critical role in food security and sustainable development since it serves as a source of genes essential in the development of well adapted and better performing varieties. To assess the genetic diversity of a specific crop, molecular (genotype) and morphological (phenotype) markers have been applied. However, morphological traits are susceptible to environmental factors, limited in number, and may vary at varied stages of development. Molecular markers have been found to be better than morphological traits since they are neutral to environmental needs and abundant in organisms. Molecular methods differ with respect to the significant features; for instance, the level of polymorphism detected, genomic abundance, technical requirements, reproducibility, locus specificity, and cost. The use of molecular markers is a strong strategy for understanding the genetics of numerous models associated with agricultural science. Breeders use these markers to provide beneficial genetic information [7].

Despite several economically important species being within this genus, few studies have been done since there are few DNA markers and familiar functional genes in this genus [8]. Most species in this genus are not major crops; thus, there are few attempts to sequence them. Transferred simple sequence repeat (SSR) markers have been used to assess *Crotalaria's* genetic diversity besides examining its germplasm's phylogenetic relationships. The first study in which polymorphic expressed sequence tag-SSR (EST-SSR) markers were obtained was reported by Wang et al. [9]. The markers were obtained from soybean and *Medicago* to assess the genetic diversity of *Crotalaria* germplasm collection. Satya et al. [6] reported the use of SCoT markers in the study of diversity among *C. pallida, C. retusa* L, *C. verrucosa* L, *C. nana, C. laburnifolia*, and *C. juncea.* The SCoT markers were not fit for the diversity assessment of some species like *C. retusa* L, although they were found to be consistent for the genetic assessment of the genus. Another study by Rather et al. [10] on the genetic diversity using ITS and *matK* gene led to the discovery of two species, namely, C. *suffruticosa* and *C. multibracteata.* The two regions would be more dependable in detecting inter- and intraspecific polymorphism in different plant species. On the other hand, transferred SSR markers have been utilized to assess genetic diversity and investigate plant germplasm's phylogenetic relationships [9]. Another effort by the group did not succeed in amplifying SSR from soybean and Medicago in *Crotalaria*, showing that sequence-specific SSR markers are essential for *Crotalaria* for microsatellite-linked genetic analysis. Diverse genetic resources provide plant breeders with a better chance of creating new and improved cultivars. Currently, agronomic trait control is believed to be regulated by multiple genes. The primary goal of creating contemporary cultivars is to identify the best genes associated with these traits. Presumably, during *Crotalaria* domestication and introduction to new areas, advantageous alleles may have been lost due to genetic bottlenecks [11]. The accessions selected for a breeding program must have and be able to transfer beneficial rare alleles absent in elite germplasm, making it essential to understand the origin of these alleles. Novel genes for a target trait are likely to be available from accessions that deviate significantly from elite genotypes [11]. Selecting accessions from the existing germplasm to employ in breeding operations is challenging. Understanding the genetic diversity of genotypes of *Crotalaria* will aid geneticists and breeders in unraveling the germplasm's structure, thus enabling them to select parents with higher genetic diversity and hastening the development of genetic resources [11].

Generally, the occurrence of SSRs or microsatellites in tropical fodder legumes has not been extensively explored, making the study of their genetic diversity challenging. The SSR markers may give more accurate results in estimating genetic diversity than other genomic markers [12]. They have been widely successfully used in the determination of genetic diversity among plant species compared to other molecular markers because of their multiple-allelic property, codominance, and relative abundance [13, 14]. The SSR markers are fundamental in pedigree analysis, genotype differentiation, assessing genetic distances among genotypes, and identification of varieties. These tandem repeats are distributed uniformly in the whole genome and have a high reproducibility as well as polymorphic information content (PIC) [15]. Besides, they differ in the polymorphism they detect based on sequence of the repeat motif they have and length, as well as their location in the noncoding or coding regions of the genome [11, 16].

Genetic diversity is the most significant aspect limiting the average number of alleles (N) identified in each SSR locus in a screening program [16]. Despite its potential economic importance, *Crotalaria* has received limited research attention; hence, there is limited information on its genetic diversity. Hence, there is need to establish its genetic diversity as a foundation for its conservation and breeding. Breeders can improve certain local varieties and enhance production by assessing regional and local plant genotypes, which is crucial in determining germplasm diversity. Since *Crotalaria* is rarely utilized in many diversity studies, breeding strategies to improve the domesticated species through interspecific hybridization can be devised with the help of diversity assessment and relationship studies. The current study aimed to optimize and validate the use of 39 designed SSR markers in characterizing and assessing the genetic diversity of 31 *Crotalaria* accessions.

## 2. Materials and Methods

### 2.1. Plant Materials

Two replicates of 31 different *Crotalaria* accessions (Table 1) obtained from different regions of Kenya as described by Muli et al. [17] were investigated in this study. The seedlings were raised under greenhouse conditions at the University of Embu Farm. In this study, all accessions with a similar domestication status were considered a population. Hence, the investigated accessions were from either domesticated or wild populations.

### 2.2. Designing the SSR Markers

An earlier study determined the gene expression profile under drought and nondrought conditions, as well as the gene expression kinetics associated with phosphorous use efficiency in *Crotalaria* accessions [18]. Different *Crotalaria* accessions were subjected to drought conditions and different phosphorous regimens. The Illumina HiSeqTM 2500 system sequenced the RNA of the treatments and the control subjects. Following the *de novo* assembly of clean reads using Trinity software (V2.0.6), SSR markers from the CDS and the unigenes were mined using the Microsatellite identification tool (MISA, http://pgrc.ipk-gatersleben.de/misa/misa.html) and then the primers for each SSR designed using Primer 3 [19]. The repeat sequence motifs entailed hexanucleotides, pentanucleotides, tetranucleotides, trinucleotides, dinucleotides, and mononucleotides. Each of these types of repeats plays a key role in determining the stability and specificity of SSR markers. A minimum repeat number of 4, 4, 5, 5, 6, and 12 was required for each type of repeat, respectively. The minimum distance between two SSRs for them to be considered distinct was set to 100 bp, while the maximum distance allowed between compound SSRs was 150 bp. To design the SSR primer pairs from the flanking sequences of the identified SSR motifs, Primer 3 an online web tool was used. For this study, a total of 39 SSR primers were randomly selected from all the designed primers. The SSR primers with the prefix D were from a drought tolerance study while those with the prefix P were from a phosphorous mobilization assay study.

### 2.3. DNA Isolation

Exactly 0.2 g of young 2-week-old leaves were carefully weighed and ground into fine powder for each *Crotalaria* accession. The total genomic DNA of each leaf sample was extracted using a modified CTAB protocol as described by Muli et al. [17]. Preventing phenolic compound and polysaccharide coprecipitation is still the greatest challenge during plant DNA extractions. Therefore, one of the modifications conducted was the addition of 30 μL of 4M Guanidinium thiocyanate (GITC) to the sample before incubation to separate the DNA from other parts of the cell, plant materials such as the polysaccharide materials, and the secondary metabolites in the recalcitrant samples, especially the wild population, and to enhance cell lysis. Besides, 50 μL of 3M sodium acetate was added before addition of the isopropanol to enhance precipitation of the DNA [20]. To the leaf powder, 500 μL of CTAB buffer (2% CTAB, 5M NaCl, 1M Tris-HCl, 0.5M EDTA, 0.2% β-mercaptoethanol, and 1% PVP) was added at room temperature and incubated at 65°C in a water bath for 30 min. Phenol:chloroform:isoamyl (25:24:1) alcohol was added to separate the DNA from other cellular substances, followed by precipitation with ice-cold isopropanol and washing with ice-cold 70% ethanol. The presence of the extracted DNA was confirmed using 1% agarose gel electrophoresis.

#### 2.3.1. Isolation of DNA From Recalcitrant Samples

Besides the CTAB protocol, the study also used the Quick-DNA Plant/Seed Kits (Zymo Research Corp) to isolate DNA from recalcitrant samples as per manufacturer instructions. This alternative approached was applied due to its ease of extraction and high-quality DNA extraction, particularly when dealing with high phenol content which is a challenging material in *Crotalaria.* All the obtained DNA was stored at −80°C while awaiting subsequent procedures.

### 2.4. Polymerase Chain Reaction (PCR) Amplification and Electrophoresis

A gradient PCR was done to determine the optimum annealing temperature for all the primers. The gradient PCR was performed to identify the optimal annealing temperature for each primer, as different sequences may bind most efficiently at different temperatures. This optimization step is crucial to ensure accurate and reproducible results across all markers. The process involved subjecting a DNA template to the PCR amplification process with varied annealing temperatures ranging from 44.4°C to 54.38°C. The PCR was performed in 25 μL reactions, containing 1 μL DNA template, 5 μL 5× standard buffer, 1 μL of 5 mM forward primer, 1 μL of 5 m reverse primer, 0.5 μL 2.5 mM dNTPs, 0.125 μL Taq DNA polymerase (New England Biolab), 2 mM magnesium chloride (MgCl_2_), and 17.375 μL nuclease-free water. The amplification was conducted in a thermocycler (Agilent Technologies SureCycler-8800 thermocycler) with the cycle profile of; initial denaturation at 94°C for 5 min, followed by 30 cycles for 40 s at 94°C, 40 s annealing at varied annealing temperature interspecific to each primer set and extension at 2 min at 72°C. The final extension proceeded at 72°C for 5 min. The primers used in the study are listed in Table 2. The resultant amplicons were analyzed using gel electrophoresis on 2% agarose gel containing 3 μL Sybbr Green at 90 V for 40 min in 1× TAE buffer. The gel wells were loaded with 3 μL DNA products mixed with 2 μL 6× DNA loading dye. A 50-bp ladder was used as a DNA size reference.

### 2.5. Genetic Analysis and Data Processing

Consistent and reproducible SSR bands were scored separately as absent (0) or present (1) in a spreadsheet for the 31 *Crotalaria* genotypes. The PIC values were calculated for the polymorphic markers PIC = 1 − ∑(pi2), where pi is the allele frequency of the *i*th allele. The genetic distance (*D*) between the two populations was estimated using the Pairwise Population Matrix of Nei's Unbiased Genetic Distance. Principal coordinate analysis (PCoA) was performed using the DARwin software 6.0.021. The PCoA model is based on distance and uses a dissimilarity matrix that is computed by a combination of factorial analysis and a simple matching index. The PCoA was conducted to evaluate the structure patterns among the 31 accessions of *Crotalaria.* Gene structure data analysis was conducted using GeneAlex software (V 6.5). DARwin and GeneAlex software were chosen for their comprehensive analysis capabilities and userfriendly interfaces. These programs are widely used for genetic diversity studies and offer robust tools for calculating genetic distance and conducting PCoA. Compared with other available software, they provide more accurate results in large datasets due to their efficient computational algorithms.

## 3. Results

### 3.1. Optimization of PCR Conditions

The results from the different primer sets showed that annealing temperatures ranging from 48.0°C to 53.49°C typically produced the best results when using 5 mM primers, 2 mM MgCl_2_, and 2.5 mM dNTP (Table 3). Primers DC009, DC010, DC012, and PC009 amplified within the temperature range (46.4°C–49.7°C). All the other SSR markers that produced single bands within the temperature ranges were selected as best primers based on clear DNA amplicons. Three primers (DC015, PU019, and PC013) produced several banding patterns of undesired sizes instead of 114, 153, and 158 bp, which were the desired sizes. Therefore, certain primers were noncompliant with optimization. Seven SSR primers (DC002, PC001, DU018, PU018, DC001, PC016, and PC007) did not amplify within the gradient temperature range tested even after optimization of the MgCl_2_ and dNTPs concentrations. These seven primers are refractory to optimization and were eliminated. Subsequently, only the remaining 29 SSR primers were used.

The annealing temperatures of the primers were determined by running a gradient PCR for the primers using one DNA sample, across the different temperature ranges (from 44.4°C to 56.0°C). All the primers were tested across various temperatures, which led to varied product intensities when observed on 2.0% agarose gel. Eight markers (DC006, DC011, DC012. PC009, PC010, PC011, PC014, and PC017) amplified well at 49.7°C. Four markers (DC004, DC005, PC004, and PU020) amplified at 48.0°C, two markers (DC017 and DU019) amplified at 48.1°C, and three markers (PC008, DC014, and DC013) amplified at 48.4°C. Only DC009 amplified well at 47.5°C. The markers DC007, PC006, PC012, and PC015 amplified at 47.8°C, DC008 amplified well at 47.3°C while DC003 amplified at 49.3°C. DC010 amplified well at 53.49°C and four markers (DC016, DU019, PC002, and PC005) amplified at 50.6°C. The optimum MgCl_2_ and dNTP concentrations were 2.0 and 2.5 mM, respectively. Finally, the PCR was conducted in a 25 μL reaction, containing 1 μL DNA template, 5 μL 5× standard buffer, 1 μL of 5 mM forward primer, 1 μL of 5 mM reverse primer, 0.5 μL of 2.5 mM dNTPs, 0.125 μL Taq DNA polymerase (New England Biolabs), 2 mM MgCl_2_, and 17.375 μL nuclease-free water (Tables 3 and 4). For all the investigated SSR markers, the optimal PCR conditions were similar, except for the annealing temperatures, which were specific to each marker as shown in Table 2.

### 3.2. Selection of the SSR Markers

The extracted plant DNA was amplified with the final optimal reaction mixture with the determined concentrations of Taq polymerase, dNTPs, and MgCl_2_ recorded in Table 3. After optimizing the PCR parameters that yielded good results, validation of the parameters was done to assess the genetic diversity in the 31 *Crotalaria* accessions. Sixteen markers were found to be polymorphic, while 13 markers produced monomorphic bands (DC007, DC008, DC009, DC012, DC013, DC014, DC017, DU019, PC002, PC005, PC006, PC012, PC017, and PC013).

### 3.3. Genetic Properties of the SSR Markers

Each of the 29 SSR markers had different abilities to determine the genetic diversity among the different *Crotalaria* accessions. A total of 89 alleles were detected from the 31 *Crotalaria* accessions using the 29 SSR markers. A total of 89 alleles were detected from the 31 *Crotalaria* accessions using the 29 SSR markers. The primers that detected the highest number (3) of alleles in the wild population were PC015, PC004, DC016 and DC011, followed by DC004, DC005, DC006, DC013, PC008, and PU020, which detected two alleles. The other primers were monoallelic. The primer DC006 detected the highest N (3) in wild population. The other primers that detected alleles include DC003 (2), DC004(2), DC005 (2), DC007 (1), DC008 (1), DC009 (1), DC010 (2), DC011 (2), DC012 (1), DC013 (1), DC014 (1), DC016 (2), DC017 (1), DU019 (1), PC003 (2), PC004 (2), PC005 (2), PC006(1), PC008(3), PC009 (2), PC010 (2), PC011 (2), PC012 (1), PC014(2) PC015 (2), PC017 (1), PU020 (2), and PC002 (1). The average N detected by the SSR markers for the wild-type population was 1.483 per locus, while within the domesticated population, the mean was 1.586 per locus. The grand mean of the N detected was 1.534 per locus. The maximum N per locus was detected by the marker PC004 which detected four (4) alleles. The number of effective alleles (Ne) detected ranged from 1.000 to 2.667, with an average of 1.197, with the marker DC013 having the highest Ne (Table S1). The observed heterozygosity (*Ho*) in the wild population was 0.070 whereas in the domesticated population, it ranged from 0.000 to 0.500 with an average of 0.097. The *Ho* values between the two populations ranged from 0.000 to 0.500, with an average of 0.083. The expected heterozygosity (*He*) for the wild population ranged from 0.000 to 0.625 with an average of 0.113, while within the domesticated population, the *He* ranged from 0.000 to 0.420 with a mean of 0.125. The total means *He* of the two populations was 0.119.

**Figure 1:**
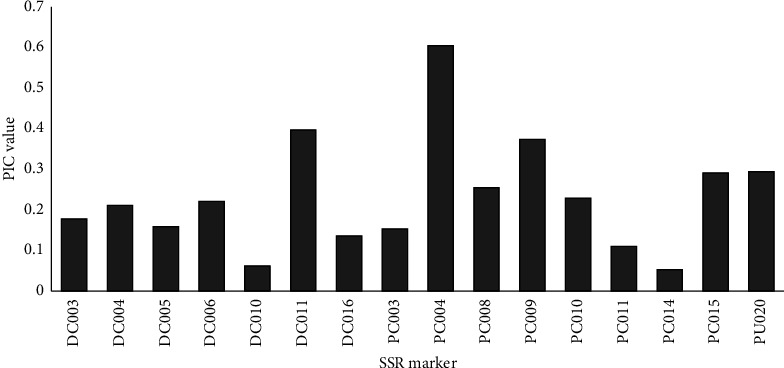
Bar graph showing the polymorphic information content of the 16 polymorphic SSR markers in 31 *Crotalaria* accessions. The values plotted on the graph represent standardized proportions of the total polymorphic markers (7 markers designed from a drought tolerance study and 9 designed from phosphorus mobilization assay study).

The mean fixation index (*F*_ST_) within the wild population was 0.234 and 0.181 for the domesticated population with a grand mean of 0.201. The average percentage of the polymorphic loci was found to be 44.83%. The domesticated population had a higher number of private alleles compared to the wild-type population. The mean number of private alleles for the wild and domesticated population was 0.345 and 0.414, respectively. Private alleles were observed in 26 different *Crotalaria* accessions (59, 121, 150, 02, 91, 217, 299, 41, GBK-5189, GBK-5199, GBK-5200, GBK-5201, GBK-5230, GBK-5244, GBK-5262, GBK-5470, GBK-5685, GBK-47581, GBK-5670, 207, 216, 233, 239, 301, GBK-5477, and GBK-5231). The PIC, which is a measure of the allelic diversity for a specific locus, ranged from 0.062 to 0.397, with a mean of 0.195 among the drought-resistant markers. For the phosphorus mobilization markers, the PIC ranged from 0.053 to 0.605, with a mean of 0.263. The marker PC004 had the maximum PIC value (0.605), while PC014 had the least value (0.053). Only one marker (PC004) had PIC value ≥ 0.50, while two markers (DC011 and PC009) had PIC values ranging between 0.30 and 0.490, and 13 markers (DC003, DC004, DC005, DC006, DC010, DC016, PC003, PC008, PC011, PC010, PU020, PC014, and PC015) having PIC values < 0.30. The PIC of all the polymorphic markers is shown in Figure 1.

Analysis of molecular variance (AMOVA) was calculated using the matrix distances for genetic differentiation. The analysis revealed that variation among individuals accounted for 87% of the variation, 9% of the variation was attributed to the differences within individuals while 4% variation was detected among the two populations (Table 5). Wright's F-statistics revealed that the FST was 0.043, showing the low genetic differentiation between the two studied populations since differences between these populations contributed 4.3% of the total variation. However, the inbreeding coefficient (FIS) of 0.909 is high, showing development of inbreeding or genetic similarity within the individuals. Moreover, the overall FIT value was 0.913, showing that, 91.3% of the variation occurs within individuals. The results showed that though a majority of the variation lies within individuals, minor but measurable differentiation exists between the populations.

### 3.4. Analysis of Genetic Diversity

**Figure 2:**
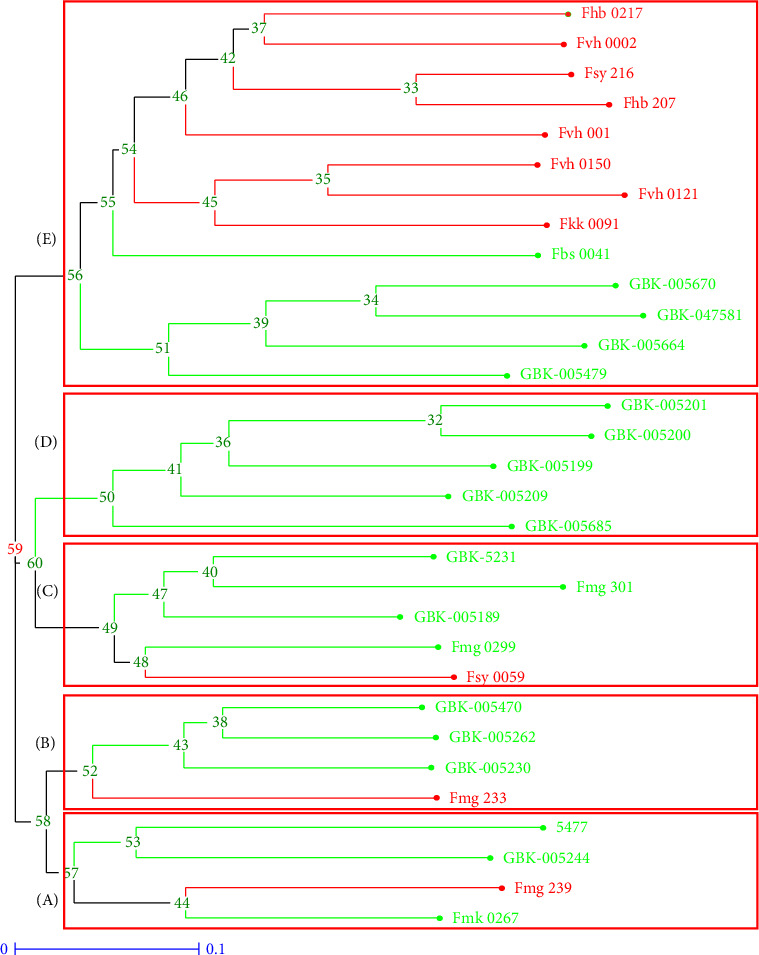
Phylogenetic tree of the 31 *Crotalaria* accessions differentiated by 29 SSR markers using the neighbor-joining method. N-J tree included five major clusters. A (four accessions), B (four accessions), C (five accessions), D (5 accessions), and E (13 accessions). Red indicates domesticated varieties, while green indicates wild varieties.

The unpaired group method of arithmetic averages (UPGMAs)-based dendrogram grouped the 31 *Crotalaria* accessions into five major clusters designated A, B, C, D and E (Figure 2). Cluster A has two subclusters. The first cluster contains accessions *C. trichotoma* Bojer (Fmg 239) and *C. lanceolate* E.Mey (Fmk 267), while the second subcluster consists of *C. incana* L. (GBK-5477) and *C. intermedia* Kotschy (GBK-5244). Cluster B has three subclusters; the first subcluster has only one accession, that is, *C. ochroleuca* G. Don (Fmg 233), while the second subcluster has accession *C. intermedia* (GBK-5230). The third subcluster has two accessions, *C. anagyroides* Kunth (GBK-5470) and *C. recta* Steud. Ex A. Rich (GBK-5262). Cluster C consists of three subclusters. The first subcluster consists of two accessions: *C. ochroleuca* (Fsy 59) and *C. deserticola* Taub. Ex Baker f. (Fmg 299). The second subcluster consists of *C. laburnifolia* L (GBK-5189), while the third subcluster has two accessions: *C. spp* (Fmg 301) and *C. intermedia* (GBK-5231). Cluster D comprises four subclusters. The first subcluster has *C. spectabilis* Roth (GBK-5685), the second one has accession *C. paulina* Schrank (GBK-5209), the third has *C. juncea* L. (GBK-5199), and the fourth one consists of *C. pancira* (GBK-5201) and *C. pallida* Aiton (GBK-5200). Cluster E has 2 subclusters. The first subcluster contains *C. brevidens* Benth var *brevidens* (Fhb 217), *C. brevidens* var *brevidens* (Fvh 0002), *C. trichotoma* (Fsy 216), *C. trichotoma* (Fhb207), (Fvh 001), *C. trichotoma* (Fvh 0150), *C. ttrichotoma* (vh 0121), *C. ochroleuca* (Fkk 0091), and *C. grahamiana* Wight & Arn (Fbs 0041). The other subcluster comprises *C. retusa* L. (GBK-005670), *C. scassellati* Chiov (GBK-047581), *C. greenwayi* Baker f. (GBK-005664), and *C. endecaphyla* (GBK-005479).

**Figure 3:**
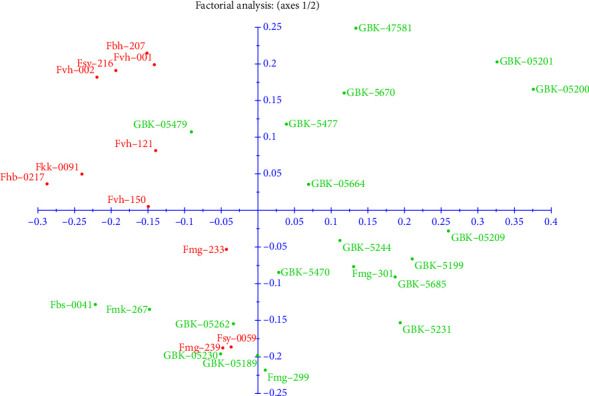
Principal coordinate analysis of 31 *Crotalaria* accessions using 29 SSR markers used to determine variation. All five coordinates have positive eigenvectors. The first two coordinates contributed to 18.13% and 11.98% in variation, respectively. Red represents domesticated accessions while green represents the wild accessions.

Based on the clustering, domesticated accessions were in similar groups, while wild accessions were generally in a different group. This shows that the domesticated varieties were closer to each other than the wild population and are more related genotypically. Domesticated accessions FSY- 0059, 239, and 233 displayed similar groupings to the wild accessions, unlike the other domesticated accessions. The PCoA (Figure 3.) shows that all accessions were distributed across the plot, with 55.27% of the total variation explained in the first five coordinates.

## 4. Discussion

### 4.1. Optimization of the SSR Markers

The annealing temperature is a crucial element that should be optimized in the PCR reaction and is determined from the denaturing temperature (Tm). The calculation is done using various formulas, and the simplest formula is the Wallace–Ikatura rule [21].

(1) $$\begin{array}{c} \text{Tm}=2\left(A+T\right)+4\left(G+C\right). \end{array}$$

*A*, *T*, *G*, and *C* are the base number of primers.

Berindean et al. [22] state that the modifications in the dNTP or the free Mg^2+^ concentration or the depletion of primer are often not considered to significantly affect the Tm. In the process of PCR amplification, the flexibility of the Tm enables optimization of the reaction in the presence of variable quantities of other ingredients (mainly the DNA template). Generally, optimal amplification relies on various factors, such as the concentration of the reagents in the buffer and the temperature profile. Optimal amplification relies on various factors, such as the concentration of the reagents in the buffer and the temperature profile. The most straightforward approach to optimizing a PCR with a specific primer pair is to modify the annealing temperature or the MgCl_2_ concentration [22]. SSR markers often show varying amplification efficiencies in PCR, making optimization crucial [23]. For uniform amplification of every SSR, extensive optimization is necessary. In this study, 2 mM MgCl_2_ was the most effective concentration for the tested primers, showing that it is the optimal concentration for the SSR markers studied in the genus *Crotalaria*. The concentration of 2 mM Mg^2+^ is within the recommended range of 1–4 mM [20]. MgCl_2_ is a significant cofactor for the DNA polymerase enzyme in PCR, and optimization of its concentration is necessary for every primer template system. Several components of the reaction bind magnesium ion including dNTPs, PCR products, templates, and primer. The Mg^2+^ bind tightly to the phosphate sugar backbone of nucleic acids and nucleotides, and differences in the concentration of MgCl_2_ has strong impacts on the interactions of nucleic acids. Differences in MgCl_2_ concentration below 4 mM enhance PCR performance by influencing specificity [23]. For instance, higher concentration leads to lower specificity while lower concentrations increase specificity.

The combination of different dNTPs and MgCl_2_ concentrations showed that 2.5 mM of dNTPs worked best with 2 mM MgCl_2_. The concentration of Taq polymerase was not optimized in this study but was maintained at the manufacturer's recommended quantity of 0.125 μL (New England Biolab). The reaction mixture was cycled several times under different temperature conditions for denaturation, annealing, and DNA synthesis. Longer durations for annealing and higher temperatures enhanced the amplification efficiency of the SSR markers. The results indicated that 30–35 cycles are adequate for a PCR reaction, suggesting that to adequately amplify the target amplicons, broad optimization is needed for the SSR PCR. This observation concurs with the results of the study conducted by Ashkani et al. [23] who reported that 30 to 35 cycles were appropriate and adequate for a good PCR reaction. Optimization was necessary to avoid shuttering, a problem of nonamplification [24]. Magnesium ions are crucial for the enzymatic activity of *Taq* polymerase. The interspecific binding is able to give the enzyme its catalytic activity or particular structure. Magnesium ions are crucial in the PCR amplification process as they influence the specificity and fidelity of any PCR reaction. The importance of Mg^2+^ in the PCR reaction depends on its concentration due to its role during the process of primer binding. Low magnesium ion concentrations may contribute to a lack of reaction due to inadequate functioning of the DNA polymerase. It may also prevent the annealing process from taking place. On the other hand, high concentrations of magnesium may render the enzyme over-reactive, resulting in nonspecific unwanted amplicons that can be detected during gel electrophoresis [23].

In the present study, the quantity of DNA strongly affected the PCR results. When 1.5 μL DNA was used, no amplification was observed. The volume of the DNA template cannot be too high as the reaction may obtain nonspecific products. Past studies have also indicated that in practice, PCR for SSR markers may fail due to several reasons. The reasons reflect several factors that could affect the amplification, including varied brands/types of thermocyclers, the reaction components (dNTPs, template DNA, concentration of the MgCl_2_, and *Taq* polymerase, among others), or even small variations in the thickness of the walls of the reaction tubes [23, 25]. Although the standard guidelines were followed during primer design, more optimization experiments involved adjusting primer length and melting temperature might have increased the amplification efficiency and specificity of the current set of primers under study. Furthermore, evaluating alternative primer sets targeting different SSRs could have yielded more successful primer sets. As such, future studies could employ in silico tools to systematically compare candidate primers based on these parameters.

The concentration of MgCl_2_ affects the success of the PCR reaction. Increasing the concentration of MgCl_2_ improves *Taq* activity to an optimum above which the Mg^2+^ might function as an inhibitor of *Taq* activity. The optimum concentration of MgCl_2_ ranges from 1.0 to 2.5 mM. As PCR substrates, dNTP content directly impacts the results of the amplification. Excess dNTP competes with the *Taq* binding to the magnesium ions, thus hindering the reaction. The quantity of *Taq* polymerase affects the efficiency of the amplification. When excess amounts of *Taq* are used, the enzyme will produce a high mismatch rate, while low amounts affect its effectiveness and primer binding [23]. Even though the primer concentration is not as crucial for the reaction as the other parameters, its optimization is economically significant to prevent unnecessary wastage. Different optimal concentrations of the reverse and forward primers of various markers have been previously reported. Generally, the concentration of the primer's ranges from 1 to 5.0 mM. Variations in the DNA sequence loci could also result in differences in the effectiveness of primer binding [23, 25]. Thus, it is vital to optimize the concentrations of the primers in order to obtain quantity products. Therefore, the obtained results from the present study propose that the concentration of all PCR components must be optimal for an effective amplification.

### 4.2. Selection and Characterization of SSR Markers in the Genus *Crotalaria*

A total of 39 pairs of SSR primers were developed to genotype 31 accessions of *Crotalaria* in the present study. Out of 39 SSR primers, 74% (29 SSR markers) successfully amplified target bands while the other 26% (10 SSR markers) did not amplify any fragments. The current study is the first study to optimize and validate SSR markers for the assessment of genetic diversity in the genus *Crotalaria.* Other studies have assessed the genetic diversity of *Crotalaria* using other molecular markers. Using the start codon targeted (SCoT) markers, Satya et al. [6] assessed the genetic diversity of 93 accessions representing seven different accessions.

The primers that produced bands with undesired sizes could bind to other parts of the genome rather than the expected sites and thereby amplify nontarget products [26]. The nonspecific binding sites of the primers are the key sources contributing to nonspecific amplifications. Cross homology in the DNA may reduce the production of the desired amplicon [27]. The failed amplification of the seven SSR markers may be possibly due to the fact that the primers were developed across large introns or splice sites [15, 28]. The outcome suggests that the primers had either sequence exon–exon junctions or were established from an incorrectly assembled transcript [29, 30]. The most abundant repeats in the present study were trinucleotide repeats, which is comparable to other studies that assessed other plants such as cereal, grape, and wheat [31, 32]. The trinucleotide ACA was the most frequently observed repeated trinucleotide in the current study. The abundance of varied motif repeats has been reported to show variable and irregular distribution in different plants. The observation that trinucleotide ACA was most frequent is unlike other studies that reported that GAA is the most abundant SSR in dicots [30].

### 4.3. Validation of the Optimized SSR Markers for Genetic Diversity Assessment in *Crotalaria*

The genetic diversity indices and allelic pattern in this study offer insight into genetic diversity within each of the two study populations. The wild population had a slightly higher *He* than the domesticated population. As a result, the wild population was more diverse compared to the domesticated population as *He* depends on both the richness (number) of alleles and the evenness (abundance) of the alleles present in a population Dagnon et al. [14]. Understanding genetic diversity with the *Crotalaria* populations offers insight and information that is crucial in monitoring and maintaining genetic diversity, which is requisite for a strong breeding program.

The development of SSR markers is a critical step in identifying genetic variation and enhancing breeding efforts in *Crotalaria* accessions. The current study lays the foundation for additional studies on genetic variation, population dynamics as well as breeding activities in the genus *Crotalaria* and other related genera. The study further sheds light on the genetic variation and breeding potential of *Crotalaria* accessions. The mean *F*_ST_ of 0.201 shows a high genetic differentiation within the studied *Crotalaria* accessions based on the Wright [33] who defined the genetic differentiation as Fst* *<* *0.05 as low, 0.05 < Fst < 0.15 as moderate and 0.15 < Fst < 0.05 as high and Fst *t* > 0.25 as very high. The *Ho* (0.083) shown by the present study is lower than the *He* (0.119) (Table S1). This information shows that there is a deficit of heterozygotes in the study populations. Generally, deficiency in heterozygotes indicates inbreeding, since inbreeding increases the number of homozygotes at the expense of heterozygotes. Lower level of heterozygosity enables the populations to adapt to their environments through different processes such as the removal of deleterious burdens and the alterations in gene expressions [34] Comparable results were obtained by Dagnon et al. [14], who assessed the genetic diversity and population structure of cowpeas using SSR markers. In their study, the authors reported an average *Ho* of 0.073. The deficit of heterozygotes observed in the present study could be a result of a moderate value of the inbreeding coefficient (FIS = 0.275). Shortage in heterozygotes in the population indicates that the population contains fewer heterozygous individuals than expected in a population at Hardy–Weinberg equilibrium. Besides, these observations could be a result of selection pressure, which might have reduced the polymorphism level in the *Crotalaria* study population. The inbreeding coefficient obtained in the present study is high (0.909). It ranges between 0.746 and 0.988 recorded by Seo et al. [35] and Fatokun et al. [36] who examined genetic diversity in cowpea accessions. The F-statistics analysis revealed that the major feature of the genetic structure in the two populations is a high degree of genetic similarity among individuals, represented by the high inbreeding coefficient. Such a high level of inbreeding can be a consequence of restricted gene flow or isolated mating within each population. Although genetic differentiation among populations is low, as expressed by the Fst value, the presence of measurable differences suggests that evolutionary processes like drift or selection still account for some population-specific genetic variation [14]. The balance between low differentiation between the populations and high variation among individuals may have implications for long-term adaptability and the potential response to environmental change in these populations. While there is a general genetic similarity, other smaller genetic differences may become magnified under different selective pressure, resulting in further divergence.

The PIC values and *He* (also referred to as gene diversity) values are both measures of genetic diversity among genotypes in the breeding populations, which highlights the evolutionary pressures on the alleles and the rates of mutation that a locus might have encountered over time [37]. The PIC values are an indication of the significance of SSR markers for linkage analysis when analyzing the inheritance between the offspring and the parental genotypes. The *He* (or gene diversity [GD]) shows gene diversity for haploid markers and offers an estimate of the genetic distance and mean heterozygosity among accessions in a population [37].

The GD of a locus, also referred to as *He*, is a key quantity of genetic diversity in a population and outlines the expected percentage of heterozygous genotypes within a population at Hardy–Weinberg equilibrium [38]. The overall *He* value in the present study was marginally higher than the PIC value, which was expected given that PIC values are always lower than *He* and converge towards *He* as allele counts increase and allele frequencies become more evenly distributed (where it is less probable that individuals have the same heterozygote genotypes). In a previous study, markers having PIC values ≥ 0.5 were described as highly informative. In contrast, those having PIC values ranging from 0.25 to 0.5 were considered to be moderately informative and those PIC < 0.25 as slightly informative [39]. In the current study, the PIC values ranged from 0.062 to 0.397, with a mean of 0.195 among the SSR markers designed to assess drought resistance. These markers could be considered to be slightly informative. For the markers designed for phosphorus mobilization, the PIC ranged from 0.053 to 0.605 with a mean of 0.263, hence could be considered as moderately informative. The grand average value for the PIC values was 0.233, which generally shows that the studied SSR markers were slightly informative in assessing the genetic diversity of the genus *Crotalaria.* The values obtained were less than the values obtained from previous studies, which reported PIC of 0.45, 0.44, and 0.58 [11, 14, 40]. The PIC is one of the most significant indicators used for the comparison of various markers of differentiation [40]. High PIC values denote a rare allele at an indicator location or high polymorphism, which plays a significant role in the differentiation of accessions. Markers that have high PIC, for instance in the present study the marker PC004 with a PIC value of 0.605, can be utilized to differentiate *Crotalaria* accessions. The frequency and N affect the values of the PIC, which is a significant indicator of marker polymorphism.

The AMOVA results revealed 87% variation among individuals within regions of the total variation, while 9% of the difference was attributed to the differences within the populations. This was unlike the study by Satya et al. [6] that revealed significant variability of 24% between the accessions and 76% within the accessions. The present study focused on intraspecific genetic differentiation within populations. Satya et al. [6] investigated both within-species and between-species genetic variance, providing a more comprehensive understanding of *Crotalaria* accessions genetic variety and differentiation. Data obtained from the analysis of the molecular variance show that there is substantial genetic diversity both within and between the populations, with some evidence of inbreeding within populations and genetic differentiation between populations. Thus, the optimized SSR markers can be applied in the genetic diversity assessment of the genus *Crotalaria* and other related genera within the family Fabaceae. The obtained results indicated that intrapopulation diversity was lower than interpopulation diversity. The low Fst value (0.043) showed a low genetic variation between the studied populations. The overall fixation and the inbreeding coefficient were 0.913 and 0.909, respectively, across the SSR loci.

### 4.4. Genetic Relationship and Population Structure

The study population was divided into two major groups based on their domestication status. The UPGMA dendrogram generated divided the studied *Crotalaria* accession into five main clusters. The groups were further subdivided into different subgroups. This clustering shows that the optimized PCR conditions for the SSR markers were successful in discriminating the 31 *Crotalaria* accessions that were evaluated. Therefore, the germplasm could be used as a valuable source for selecting diverse parents for a breeding program aimed at developing new cultivars with different traits of interest.

Selecting and creating plant cultivars with desired characteristics is a common step in the domestication process. This in turn reduces the genetic diversity compared with wild counterparts. Genetic divergence and local adaptations result from natural selection in several settings. Geographic isolation increases genetic distinctiveness by further limiting gene flow between groups. The high genetic distinctiveness observed might also be attributed to significant changes in allele frequencies over time brought about by genetic drift in small, isolated wild populations. Despite them belonging to different accessions, breeders can take advantage of their traits to improve domesticated varieties during breeding programs. Phenotypically, the varieties in the fifth subcluster were different. However, from the genotypic analysis, they seem to be closely related, hence can be improved genetically. The PCoA results showed that the *Crotalaria* accessions could be grouped into four clusters and this concurred with the results of the neighbor-joining tree analysis.

Like many plants, *Crotalaria* accessions are mostly identified using their common names and their morphological features such as pod shape, flower morphology, seed size, and color as well as leaf structure. However, there are drawbacks to morphological characterization, including limited variability between accessions due to the limited genetic base of the germplasm used, the influence of the environment on the expression of the characters, and the length of time required to obtain results [41]. Besides, this nomenclature can result in the redundancy of some accessions in the germplasm collection. Most studies have determined that SSRs are appropriate markers for assessing genetic diversity, Quantitative Trait Loci (QTL) studies and the population structure of most plants in the family Fabaceae. For instance, Dagnon et al. [14] successfully assessed the genetic diversity of cowpeas using SSR markers.

## 5. Conclusion

The present study reports the first successful optimization and validation of SSR markers for the assessment of genetic diversity in *Crotalaria.* Twenty nine out of the 39 optimized primers were successful. The current study provides crucial information on the ability of these markers to discriminate *Crotalaria* accessions as well as other members in the family Fabaceae. The SSR markers employed in this study were found to be slightly informative. The SSR marker PC004 detected the largest N despite being a hexanucleotide motif repeat. Its uniqueness makes it a noteworthy marker, which augments its use in the assessment of genetic diversity. By leveraging this marker, researchers can develop insight into the evolutionary mechanism and genetic patterns that shape biological diversity across various populations and species. These primers will be useful in the analysis and assessment of genetic diversity and population structure of the genus *Crotalaria*, screening of the germplasm and selection.

## Figures and Tables

**Table 1:** Domesticated and wild *Crotalaria* accessions used in optimization and validation of SSR markers in the assessment of the genetic diversity.

S/N	*Crotalaria* spp	Sample number	Domestication status
1	*C. brevidens* Benth var *intermedia*	FVH-001	Domesticated
2	*C. brevidens* var *brevidens*	FVH-002	Domesticated
3	*C. brevidens* var *brevidens*	FHB-0217	Domesticated
4	*C. ochroleuca* G. Don	FSY-0059	Domesticated
5	*C. ochroleuca*	FKK-0091	Domesticated
6	*C. ochroleuca*	FMG-233	Domesticated
7	*C. trichotoma* Bojer	FVH-121	Domesticated
8	*C. trichotoma*	FVH-150	Domesticated
9	*C. trichotoma*	FMG-239	Domesticated
10	*C. trichotoma*	FSY-216	Domesticated
11	*C. trichotoma*	FHB-207	Domesticated
12	*C. lanceolata* E. Mey	FMK-267	Wild
13	*C. recta* Steud.ex A. Rich	GBK-05262	Wild
14	*C. paulina* Schrank	GBK-05209	Wild
15	*C. pancira*	GBK-05201	Wild
16	*C. juncea* L	GBK-5199	Wild
17	*C. greenwayi* Baker f.	GBK-05664	Wild
18	*C. pallida* Aiton	GBK-05200	Wild
19	*C. laburnifolia* L.	GBK-05189	Wild
20	*C. endecaphyla*	GBK-05479	Wild
21	*C. anagyroides* Kunth	GBK-5470	Wild
22	*C. grahamiana* Wight & Arn	FBS-0041	Wild
23	*C. spectabilis* Roth	GBK-5685	Wild
24	*C. deserticola* Taub. Ex Baker f.	FMG-299	Wild
25	*C. intermedia* Kotshcy	GBK-05230	Wild
26	*C. intermedia*	GBK-5244	Wild
27	*C. intermedia*	GBK-5231	Wild
28	*C. spp*	FMG-301	Wild
29	*C. incana* L.	GBK-5477	Wild
30	*C. retusa* L.	GBK-5670	Wild
31	*C. scassellatii* Chiov	GBK-47581	Wild

*Note:* The *Crotalaria* accessions and their respective sample numbers are listed, along with their domestication status. The domesticated and wild accessions were selected to examine the genetic variability within different cultivation environments.

**Table 2:** Primer information of selected SSR markers optimized for diversity studies in *Crotalaria.*

Srl. no.	SSR marker	Forward primer (5′-3′)	Reverse primer (5′-3′)	Product size	Annealing temperature (°C)
1	DC003	CATGAACACACATCACAGTTCCT	CTTCTCCGTTTGAGAGCAAGTAA	107	49.3
2	DC004	GTTAAAGTTGTCACCCATGTTCC	TCGGTGGTTTGAATATGTTTTTC	136	48
3	DC005	GTGCTCAAGAAGTGTGGGATCTA	CAGTATGATATCACGCGAAGGAT	156	48
4	DC006	AATCAGCTTGAATCATCAACGTC	GGTTTGGTTACTCTGCCATGTAG	132	49.7
5	DC007	AATACTTGTTTGCTTCAAATGCC	AGTCAAGGTCTTTGATCTCGATG	108	47.8
6	DC008	TCATAAGGGAAATGAAACAGGAA	ATTCAATCCTGATCTTCCTCCAC	155	47.3
7	DC009	AACCTCTCCTCTTCTCCATCATC	AAGAAGGATCTTTTTGGGTTTTG	127	47.5
8	DC010	CTTGCTACACGAAAACTGTCAGA	TGTGTCGTGGTTCAATACAGTTC	132	53.49
9	DC011	GAGTGAGCTATGGTTTCTCCAAA	TGGCCTATTTTAGTGGCATTGTA	137	49.7
10	DC012	CGGAGAATTTCCACTTGATACA	TTCAGCGAAAACCCTACTCACTA	131	49.7
11	DC013	CCTCCATCTACCATCTCTGCTT	AAGTCAATGGAGAGCAATTTGAG	156	48.4
12	DC014	CTGGTCGGAGTTTGTATTTGAAC	TTCTTCACTTCCTCACCTCTCTG	142	48.4
13	DC016	GACACCACAGAGTCAAAGATGTG	ATAGTTTTCTGGGAAAGTTTGGG	150	50.6
14	DC017	ATTAGCATTTCATCACCTTCACC	TTTTGGATCTGATTTTGGAATTG	154	48.1
15	DU019	TCATATGGAAATATTTTATTGGAAA	CACGAAGAAGAAGGGTTTGCT	235	48.1
16	PC002	TGGATAGTAGCAAGGGTGCTAAA	CACTCTCTCCGACTGAAGCTAAA	160	50.6
17	PC003	CCTCCACCTCTTTCTTCACTGAC	AATTTGATTTGTTTGCTGCAGTT	139	50.6
18	PC004	ATACTCATTTTCTGTGGAAGCCA	TTTATTGCAGCAAGAGAAGATCC	96	48
19	PC005	AGATCAGATTCTTGCAAGCTCAC	ATTGCCAACTTTAACTCCTCTCC	129	50.6
20	PC006	AACCACCAAACAAACACCACTT	TCGATTGTCCACGTCTAATTTCT	157	47.8
21	PC008	CTCGTATTTCTCACAAACCCAAC	ACCATATGGGGAGTTAGAAGAGC	89	48.4
22	PC009	TACTCCATTCATTCACCACCTTT	TTGATCTCTTCAAGGCTGATACC	83	49.7
23	PC010	ACACCATAGCTTTTTCTTCCACA	AGGGAGCGAGAATCATAGCTAAC	147	49.7
24	PC011	CGCTAGAGTGCCATAATCAAATC	ATTTGGAAATAGGAAATTATAGTAACA	127	49.7
25	PC012	GTGTCAGGTACGAAATCTGGAAA	CCCGTTTACTTGTTGTTTGCTTA	154	47.8
26	PC014	GGTCTCTTTCTTCTCCATCCACT	GAATTGGAAACCCTAACAACGAT	154	49.7
27	PC015	TTTCATCAGCAGTTCAACACAAT	GGGGGATTGAAACTTGATGTATT	152	47.8
28	PC017	CTAACTCCACCAAGTTTGCTGTT	ATCAAGCCCTTATCTTTCTCAGC	136	49.7
29	PU020	TCGCTTTTAACCATCACTGAAAT	GAGGAAGAGTTGAAATTGAGGGT	116	48.0

*Note:* Markers were chosen based on amplification efficiency, specificity, and polymorphism potential.

**Table 3:** The concentrations and quantities of final optimized PCR conditions for 29 SSR primers in 25 μL PCR reaction mix for the analysis of genetic diversity among the 31 *Crotalaria* accessions.

Component	Concentration	Volume (μL)
DNA template		1
Taq standard buffer	5×	5
DNTPs	2.5 mM	0.5
Forward primer	5 mM	1.25
Reverse primer	5 mM	1.25
*Taq* polymerase		0.125
MgCl_2_	2 mM	0.75
H_2_O		15.125

**Table 4:** Summary of PCR optimization for the SSR primers. Amplification did not occur at 1.0 and 2.5 mM MgCl_2_ and 5.0 and 10 mM dNTPs concentrations.

Primer	Product size (bp)	Annealing temp (°C)	MgCl_2_ (mM)	dNTPs (mM)	Amplification result
DC012	131	49.7	2.0	2.5	Single clear band
DC013	156				
PC017	136				

DC006	132	49.7	2.0	2.5	Multiple clear bands
DC011	137				
PC009	83				
PC010	147				
PC011	127				
PC014	154				

DC004	136	48.0	2.0	2.5	Multiple clear bands
DC005	156				
PC004	96				
PU020	116				

DC017	154	48.1	2.0	2.5	Single clear band
DU019	235				

DC014	142	48.4	2.0	2.5	Single clear band

PC008	89	48.4	2.0	2.5	Multiple clear bands

PC002	160	50.6	2.0	2.5	Single clear bond
PC005	129				

PC003	139	50.6	2.0	2.5	Multiple clear bands
PC004	96				

DC010	139	53.49	2.0	2.5	Multiple clear bands

DC007	108	47.8	2.0	2.5	Single clear band
PC006	154				

PC012	154	47.8	2.0	2.5	Multiple clear bands
PC015	152				

DC015	—	Varying from 48.0 to 53.49 (gradient)	—	—	Several banding patterns of undesired sizes instead of 114, 153, and 158 bp, which were the desired sizes
PU019					
PC013					

DC002, PC001, DU018, PU018, DC001, PC016, PC007	—	Varying from 48.0 to 53.49 (gradient)	—	—	No amplification

**Table 5:** Analysis of molecular variance (AMOVA) based on 29 SSR markers studied in the genus *Crotalaria*.

Source	Df	SS	MS	Est. var.	%
Among Pops	1	22.098	22.098	0.312	4
Among Indiv	29	383.886	13.237	6.304	87
Within Indiv	31	19.500	0.629	0.629	9
Total	61	425.484		7.245	100

*Note:* % = percent variation.

Abbreviations: df = degree of freedom, Est. var = estimated variance, SS = sum of squares.

## Data Availability

All the data generated or analyzed during this study are included within the article and its Additional files.

## Supporting Information

Table S1: Genetic analysis information carried out using GeneAlex Software (v 6.5) showing N, Ne, information index (I), *Ho*, *He*, and unbiased heterozygosity (uHe).
